# Frontiers in Integrative Genomics and Translational Bioinformatics

**DOI:** 10.1155/2015/725491

**Published:** 2015-10-28

**Authors:** Zhongming Zhao, Victor X. Jin, Yufei Huang, Chittibabu Guda, Jianhua Ruan

**Affiliations:** ^1^Department of Biomedical Informatics, Vanderbilt University School of Medicine, Nashville, TN 37203, USA; ^2^Department of Psychiatry, Vanderbilt University School of Medicine, Nashville, TN 37212, USA; ^3^Department of Cancer Biology, Vanderbilt University School of Medicine, Nashville, TN 37232, USA; ^4^Department of Molecular Medicine, The University of Texas Health Science Center at San Antonio, San Antonio, TX 78229, USA; ^5^Department of Electrical and Computer Engineering, The University of Texas at San Antonio, San Antonio, TX 78249, USA; ^6^Department Genetics, Cell Biology and Anatomy, University of Nebraska Medical Center, Omaha, NE 68198, USA; ^7^Department of Computer Science, The University of Texas at San Antonio, San Antonio, TX 78249, USA

As we have officially entered the big data era, we are embracing numerous opportunities but also meeting strong demand on innovative approaches to managing and analyzing the data in the digital world. The speed of data generation is astonishing—from now until 2020, the volume of the digital data is expected to approximately double every two years, and 90% of the data available to us today were generated in just the last two years. Specifically in biological and biomedical research fields, we have witnessed the rapid advances in biotechnologies, especially the next-generation sequencing and single cell technologies, enabling the investigators to create massive amounts of data for genomics and translational research. While analysis of the data from single domain like mutations or gene expression is often the first choice in a project, integrative genomic approaches have more advantages such as robustness in detecting the biological signals or biomarkers and low false discovery rates due to the evidence from multiple domains. Therefore, we have steadily seen more integrative genomic studies during the past several years. Although these approaches are promising and powerful, there are many challenges that are required to be addressed by bioinformaticians, computational biologists, and other scientists. These challenges include, but are not limited to, data quality and process from different technology platforms, sample size and consistency, data missingness, incomplete and inaccurate knowledgebase (e.g., reference networks and pathways), false discovery, lack of novel algorithms for data integration, computational efficiency, data interpretation, and visualization.

Translational bioinformatics is an emerging field that focuses on applying informatics methodology to the increasing amount of biomedical and genomic data in order to generate knowledge for clinical applications. With the large genomic data linked to phenotype and medical records, we now can not only discover interesting biological features and regulations using genomic approaches, but also translate some of the findings for clinical practice. For example, investigators have been interested in finding actionable mutations that can be used for development of precision medicine strategies from thousands of mutations or even more in an individual genome. In addition to the challenges above, there are other topics that require immediate attention such as ownership and privacy of the findings, data sharing, efficient clinical decision support system, and design and development of specific gene panel for fast patient screening, among others.

Therefore, we launched this special issue to address the demand for integrative genomics and translational bioinformatics. We are interested in both new algorithms/tools and applications. The special issue welcomes the genomics, bioinformatics, and computational work in broad areas such as various omics technologies, multidimensional data integration, systems biology approaches, precision medicine studies, single cell research, pharmacogenomics, machine learning, high performance computing, and visualization. Special call for papers went through The International Conference on Intelligent Biology and Medicine (ICIBM 2014, held on December 4–6, 2014, http://compgenomics.utsa.edu/icibm2014/) and* BioMed Research International* journal website. After rigorous peer review, articles were selected for this special issue. We briefly describe the research projects presented in these articles as follows.

Three papers present the work to advance the next-generation sequencing technologies. In “A Comparison of Variant Calling Pipelines Using Genome in a Bottle as a Reference,” A. Cornish and C. Guda performed a systematic evaluation of variant callers to determine which pipeline has the best performance in variant calling. They compared six different aligners and five different variant callers—a total of 30 combinations—using the data generated by NIST Genome in a Bottle Consortium. For single nucleotide variant call, the authors found that Novoalign combined with GATK UnifiedGenotyper had the highest sensitivity while keeping a low false positive rate. However, calling insertion and deletion (indel) variants still remained a big challenge—none of the tools could achieve an average sensitivity higher than 33% or a positive predictive value (PPV) higher than 53%. In the paper entitled “RNAseq by Total RNA Library Identifies Additional RNAs Compared to Poly(A) RNA Library,” Y. Guo et al. evaluated the ability of detecting RNA for two popularly used RNA libraries in RNA sequencing: the poly(A) captured RNA library, which captures RNA based on the presence of poly(A) tails at the 3′ end, and the total RNA library, which captures total RNA. By using the two breast cancer cell lines, the authors found that the RNA expression values captured by both RNA libraries were highly correlated, but the number of RNA molecules captured by the total RNA library was significantly higher than that by the poly(A) library. The authors also identified several specific RNA sets that could not be captured by the poly(A) library. In the paper entitled “Assessing Computational Steps for CLIP-Seq Data Analysis,” Q. Liu et al. presented a systematic evaluation of major computational steps for identifying RNA-binding protein (RBP) using a special technology: CLIP-Seq. CLIP (cross-linking and immunoprecipitation) is designed to study protein-RNA interactions* in vivo*, such as RNA and RBP interactions. The authors evaluated data analysis steps including preprocessing, selection of control samples, peak normalization, and motif discovery. The authors reported three factors (avoiding PCR amplification artifacts, normalizing input RNA or mRNAseq, and defining the background model from control samples) could help reduce the bias due to the RNA abundance and could improve detecting binding sites. The work is helpful for analysis of CLIP-Seq data.

Cancer is a common complex disease and can occur in many tissue types and different parts of the body. Molecular data may be useful to classify cancer sites or subtypes. In the paper “Classification of Cancer Primary Sites Using Machine Learning and Somatic Mutations,” Y. Chen et al. attempted to classify cancer primary sites using large-scale somatic mutations observed in cancer genomes and machine learning method. Specifically, they examined the patterns of 1,760,846 somatic mutations identified from 230,255 cancer patients covering 17 tumor sites using support vector machine (SVM). Through a multiclass classification experiment and using gene symbol, somatic mutation, chromosome, and gene functional pathway as predictors, the authors reported the performance of the baseline using only gene features to be 0.57 in accuracy, but it was improved to 0.62 when adding the information of mutation and chromosome. Moreover, F-measure values could reach 0.70 in five primary sites with the large intestine being 0.87. The study suggested that the somatic mutation information is useful for prediction of primary tumor sites. In another machine learning paper, entitled “Construction of Pancreatic Cancer Classifier Based on SVM Optimized by Improved FOA,” H. Jiang et al. introduced an improved quantum fruit fly optimal algorithm (FOA) based method. Specifically, the improved FOA was used to optimize the parameters of SVM and a classifier was constructed based on the optimized SVM. The authors applied their method to classify pancreatic cancer and showed improved performance.

Systems pharmacology has emerged as a major computational field, which systems biology approaches have often applied to large-scale complex drug data. This special issue includes two papers in this area. In the paper “How to Choose In Vitro Systems to Predict In Vivo Drug Clearance: A System Pharmacology Perspective,” L. Wang et al. evaluated the performance of different recombinant human enzyme expression systems for predicting hepatic clearance in human body. The performance of different* in vitro* systems was compared after* in vitro*-*in vivo* extrapolation. Among the four systems (*Escherichia coli* system, yeast system, lymphoblastoid system, and baculovirus system) they compared, baculovirus system had the best performance and was suggested to be the most suitable system for the large-scale drug clearance prediction. In the paper entitled “Predicting Drug-Target Interactions via Within-Score and Between-Score,” J. Y. Shi et al. presented their computational prediction of drug-target interactions (DTIs). They characterized each drug-target pair (DTP) as a feature vector of within-scores and between-scores so that their approach has consistent form of DTPs, a reduced bias, and sharing the same visualized space between known DTIs and unapproved DTPs. They evaluated the effectiveness of their approach by comparing with other popular methods under cross-validation and predicting potential interactions for DTPs under the validation in existing databases.

In the paper “Coexpression Network Analysis of miRNA-142 Overexpression in Neuronal Cells,” I. Thapa et al. applied a correlation network model to find the coexpressed genes and how miRNA-142 overexpression impacts on the network. The authors focused on miRNA-142 because it was found to be upregulated in neurons and its overexpression plays important roles in other genes like* SIRT1* and* MAOA*. They found that several nervous system development related genes such as* TEAD2*,* PLEKHA6*, and* POGLUT1* were affected by miRNA-142 overexpression.

In the paper “OperomeDB: A Database of Condition-Specific Transcription Units in Prokaryotic Genomes,” K. Chetal and S. C. Janga present OperomeDB, a database that ensembles all the predicted operons for bacterial genomes using available RNA-sequencing datasets across a wide range of experimental conditions. The database currently contains nine bacterial organisms and 168 transcriptomes from which operons were predicted by the authors. Web interface, visualization, data query, and other functions are provided.

In the paper entitled “Building Integrated Ontological Knowledge Structures with Efficient Approximation Algorithms,” Y. Xiang and S. C. Janga tackled a basic problem on integrating a pair of ontology tree structures with a given closeness matrix. After they identified optimal structures for the problem, the authors proposed optimal and efficient approximation algorithms for integrating a pair of ontologies as well as multiple ontologies. Their results using Gene Ontology and National Drug File Reference Terminology suggested that the method should be effective on association studies between biomedical terms.

